# Large Language Models in Dental Licensing Examinations: Systematic Review and Meta-Analysis

**DOI:** 10.1016/j.identj.2024.10.014

**Published:** 2024-11-12

**Authors:** Mingxin Liu, Tsuyoshi Okuhara, Wenbo Huang, Atsushi Ogihara, Hikari Sophia Nagao, Hiroko Okada, Takahiro Kiuchi

**Affiliations:** a Department of Health Communication, Graduate School of Medicine, The University of Tokyo, Bunkyo, Tokyo, Japan; b Department of Health Communication, School of Public Health, Graduate School of Medicine, The University of Tokyo, Bunkyo, Tokyo, Japan; c Department of Clinical Epidemiology and Health Economics, School of Public Health, The University of Tokyo, Bunkyo, Tokyo, Japan; d Faculty of Human Sciences, Waseda University, Tokorozawa, Japan

**Keywords:** Dentistry, Systematic review, Oral medicine, Dental education, Healthcare

## Abstract

**Introduction and aims:**

This study systematically reviews and conducts a meta-analysis to evaluate the performance of various large language models (LLMs) in dental licensing examinations worldwide. The aim is to assess the accuracy of these models in different linguistic and geographical contexts. This will inform their potential application in dental education and diagnostics.

**Methods:**

Following Preferred Reporting Items for Systematic Reviews and Meta-Analyses guidelines, we conducted a comprehensive search across PubMed, Web of Science, and Scopus for studies published from 1 January 2022 to 1 May 2024. Two authors independently reviewed the literature based on the inclusion and exclusion criteria, extracted data, and evaluated the quality of the studies in accordance with the Quality Assessment of Diagnostic Accuracy Studies-2. We conducted qualitative and quantitative analyses to evaluate the performance of LLMs.

**Results:**

Eleven studies met the inclusion criteria, encompassing dental licensing examinations from eight countries. GPT-3.5, GPT-4, and Bard achieved integrated accuracy rates of 54%, 72%, and 56%, respectively. GPT-4 outperformed GPT-3.5 and Bard, passing more than half of the dental licensing examinations. Subgroup analyses and meta-regression showed that GPT-3.5 performed significantly better in English-speaking countries. GPT-4’s performance, however, remained consistent across different regions.

**Conclusion:**

LLMs, particularly GPT-4, show potential in dental education and diagnostics, yet their accuracy remains below the threshold required for clinical application. The lack of sufficient training data in dentistry has affected LLMs’ accuracy. The reliance on image-based diagnostics also presents challenges. As a result, their accuracy in dental exams is lower compared to medical licensing exams. Additionally, LLMs even provide more detailed explanation for incorrect answer than correct one. Overall, the current LLMs are not yet suitable for use in dental education and clinical diagnosis.

## Introduction

### Background

Since the public release of OpenAI's large language model (LLM) chatbot, ChatGPT, in November 2022,^1^ LLMs have achieved remarkable success in various fields, including medicine, law, finance, and programming.^2^, ^3^, ^4^, ^5^ Researchers also explored the potential roles and impact of LLMs in the field of dentistry.^6^, ^7^, ^8^, ^9^, ^10^ First, previous studies suggested that LLMs could be used to analyse patient symptoms and suggest potential diagnoses in telemedicine for dental clinical diagnoses.^6^^,^^7^^,^^9^ Particularly in remote areas lacking dental services, LLMs can be utilized to monitor patients’ dental health and hygiene, providing regular reminders and assessments.^9^ Second, Given their extensive datasets, LLMs also have the potential to serve as clinical decision support tools.^7^^,^^9^ For example, LLMs possess superior treatment planning capabilities.^10^ They can easily and automatically document large volumes of digital information related to healthcare. In the past, such data analysis required significant human effort. However, with the natural language reasoning capabilities of LLMs, they can interpret this data, assisting dentists in more effectively developing treatment plans tailored to the patient's background.^10^ Third, LLMs could be utilized to provide dental health information to patients.^6^^,^^9^ LLMs can also impart dental knowledge to students and professionals.^9^ Notably, compared to traditional search engines such as Google, LLMs provide more concise and direct answers, allowing users to bypass the process of filtering information.^7^ Previous study suggested that LLMs are gradually replacing search engines, as users increasingly prefer to obtain healthcare information through LLMs.^7^ Therefore, they play a role in dental education for both patients and dental professionals. Fourth, most advanced LLMs have the capability to analyse images. It offers the potential for LLMs to serve as a ‘virtual mentor’, capable of analysing dental images such as X-rays.^10^ In the past, dentists often needed to invest significant time and effort in analysing X-ray images and CBCT to diagnose dental conditions.^10^ LLMs can effectively assist dentists in analysing images to diagnose various dental diseases, including orthodontics, restorative dentistry, oral implantology, and oral and maxillofacial surgery.^10^

However, the accuracy of dental knowledge held by LLMs is a critical prerequisite for their application in both dental clinical diagnostics and education.

One of the most common methods for evaluating the accuracy of dental knowledge possessed by LLMs is to input questions from dental licensing examinations and score the responses generated by the LLMs.^11^, ^12^, ^13^, ^14^, ^15^, ^16^, ^17^, ^18^, ^19^, ^20^, ^21^ However, the performance of LLMs varies depending on the testing language, examination format, and the country where the examination originates. A study using the 2022 Certification in Implant Dentistry examination found that GPT-4 outperformed human dentists and passed the test.^15^ In contrast, A Japanese study reported that GPT-4 did not pass Japanese National Dentist Examination.^11^ These conflicting results are perplexing and make it difficult to ascertain the true accuracy of LLMs’ knowledge in the field of dentistry. Moreover, an overabundance of positive experimental outcomes might lead dental practitioners to develop a biased view of LLMs’ capabilities. This could potentially lead to the irresponsible and premature application of these models in clinical diagnostics, which could have serious consequences. To the best of our knowledge, no study has yet provided a fair and comprehensive evaluation of LLMs’ performance in dental examinations.

### Study aims and objectives

By reviewing previous studies, our research aims to comprehensively evaluate the performance of LLMs in dental licensing examinations, filling the gap in the application of LLMs in dental licensing examinations. We synthesize the findings from previous studies to offer a thorough and impartial perspective. We also explore potential factors that may influence LLMs’ performance and identify their flaws and limitations. The insights gained from our study will help dental practitioners and researchers better understand the capabilities of LLMs in the field of dentistry. This will enable more effective and judicious use of AI tools in both dental clinical diagnostics and education.

## Material and methods

Our systematic review followed the Preferred Reporting Items for Systematic Reviews and Meta-Analyses flow diagrams and guidance.^22^ We registered this study in International Prospective Register of Systematic Reviews database on 1 May 2024. The registration number was CRD42024542252.

### Search strategy

We utilized the advanced search features of PubMed, Web of Science (WOS), and Scopus to search for specific query strings (Table 1). As ChatGPT was the first LLM released to public in November 2022, our literature search included publications from 1 January 1 2022 to 1 May 2024. The literature obtained from these three databases was imported into Rayyan. Two authors (ML and WH) independently reviewed the titles and abstracts of the identified studies using a predefined search strategy to determine their compliance with the inclusion and exclusion criteria (Table 2). The full texts of these studies were then retrieved and evaluated for eligibility by the same two authors independently. Any disagreements about the eligibility of particular studies were resolved through discussion with a third reviewer (TO). During the screening process, we documented the reasons for excluding studies and presented these in a Preferred Reporting Items for Systematic Reviews and Meta-Analyses flow diagram.

**Table 1:** Query strings of WOS, Scopus, and PubMed.

WOS	TS = (("ChatGPT" OR "GPT" OR "Generative pre-trained transformer" OR "Gemini" OR "Bard" OR "Claude" OR "Copilot" OR "Bing" OR "AI" OR "artificial intelligence" OR "chatbot*" OR "large language model*" OR "LLM") AND ("dental licensing exam*" OR "dental exam*" OR "dental" OR "dentist exam*" OR "dentist" OR "dentistry exam*" OR "dentistry" OR "endodontics" OR "orthodontics" OR "periodontics" OR "prosthodontics" OR "oral" OR "tooth" OR "medical licensing exam*" OR "medical education" OR "licensing" OR "license" OR "exam" OR "exams" OR "examination")) OR TI = (("ChatGPT" OR "GPT" OR "Generative pre-trained transformer" OR "Gemini" OR "Bard" OR "Claude" OR "Copilot" OR "Bing" OR "AI" OR "artificial intelligence" OR "chatbot*" OR "large language model*" OR "LLM") AND ("dental licensing exam*" OR "dental exam*" OR "dental" OR "dentist exam*" OR "dentist" OR "dentistry exam*" OR "dentistry" OR "endodontics" OR "orthodontics" OR "periodontics" OR "prosthodontics" OR "oral" OR "tooth" OR "medical licensing exam*" OR "medical education" OR "licensing" OR "license" OR "exam" OR "exams" OR "examination")) OR AB = (("ChatGPT" OR "GPT" OR "Generative pre-trained transformer" OR "Gemini" OR "Bard" OR "Claude" OR "Copilot" OR "Bing" OR "AI" OR "artificial intelligence" OR "chatbot*" OR "large language model*" OR "LLM") AND ("dental licensing exam*" OR "dental exam*" OR "dental" OR "dentist exam*" OR "dentist" OR "dentistry exam*" OR "dentistry" OR "endodontics" OR "orthodontics" OR "periodontics" OR "prosthodontics" OR "oral" OR "tooth" OR "medical licensing exam*" OR "medical education" OR "licensing" OR "license" OR "exam" OR "exams" OR "examination")) OR AK = (("ChatGPT" OR "GPT" OR "Generative pre-trained transformer" OR "Gemini" OR "Bard" OR "Claude" OR "Copilot" OR "Bing" OR "AI" OR "artificial intelligence" OR "chatbot*" OR "large language model*" OR "LLM") AND ("dental licensing exam*" OR "dental exam*" OR "dental" OR "dentist exam*" OR "dentist" OR "dentistry exam*" OR "dentistry" OR "endodontics" OR "orthodontics" OR "periodontics" OR "prosthodontics" OR "oral" OR "tooth" OR "medical licensing exam*" OR "medical education" OR "licensing" OR "license" OR "exam" OR "exams" OR "examination"))
Scopus	TITLE-ABS-KEY (("ChatGPT" OR "GPT" OR "Generative pre-trained transformer" OR "Gemini" OR "Bard" OR "Claude" OR "Copilot" OR "Bing" OR "AI" OR "artificial intelligence" OR "chatbot*" OR "large language model*" OR "LLM") AND ("dental licensing exam*" OR "dental exam*" OR "dental education" OR "dental" OR "dentist exam*" OR "dentist" OR "dentistry exam*" OR "dentistry" OR "endodontics" OR "orthodontics" OR "periodontics" OR "prosthodontics" OR "oral" OR "tooth" OR "medical licensing exam*" OR "medical education" OR "licensing" OR "license" OR "exam" OR "exams" OR "examination"))
PubMed	("ChatGPT" OR "GPT" OR "Generative pre-trained transformer" OR "Gemini" OR "Bard" OR "Claude" OR "Copilot" OR "Bing" OR "AI" OR "artificial intelligence" OR "chatbot*" OR "large language model*" OR "LLM") AND ("dental licensing exam*" OR "dental exam*" OR "dental education" OR "dental" OR "dentist exam*" OR "dentist" OR "dentistry exam*" OR "dentistry" OR "endodontics" OR "orthodontics" OR "periodontics" OR "prosthodontics" OR "oral" OR "tooth" OR "medical licensing exam*" OR "medical education" OR "licensing" OR "license" OR "exam" OR "exams" OR "examination")

**Table 2:** Inclusion and exclusion criteria.

Inclusion criteria	Exclusion criteria
1. The study tested the performance of LLMs in dentistry-related (eg, dentistry, endodontics, orthodontics) licensing examinations 2. Any type of publications (peer-reviewed articles, conferences articles, preprints, letters, books, etc.) 3. Literature published from 1 January 2022 to 1 May 2024 4. Publications of LLMs tested in all languages 5. Studies that tested any LLMs	1. Examinations other than national dental licensing examination (eg, medical licensing examinations, nursing licensing examinations, dentistry final examinations at universities, dental questions created by the authors themselves) 2. Duplicate studies 3. Studies that are not published in English 4. Systematic review

### Data extraction and management

Two reviewers (ML and WH) independently extracted data from the included studies into an Excel spreadsheet. Any inconsistencies were resolved through consensus or by a third reviewer (TO). The general characteristics extracted included the following: (1) title, (2) authors, (3) year of publication, (4) publication date, (5) type of publication, (6) country of the dental licensing examination, (7) name of the dental licensing examination, (8) LLMs, (9) language in which LLMs was tested, (10) duration of the test, (11) type of questions, (12) number of correct or total questions, (13) accuracy rate, (14) whether the LLMs pass the examination, (15) comparison with medical students, and (16) whether a prompt used.

### Assessing the risk of bias in the included studies

The evaluation framework employed in this study was developed based on Quality Assessment of Diagnostic Accuracy Studies-2 (QUADAS-2).

QUADAS-2 evaluates the risk of bias and applicability of systematic review across four key domains: patient selection, index test, reference standard, and flow and timing. Each domain includes signalling questions to help identify potential sources of bias, such as whether patient selection was appropriate, whether the index test was influenced by knowledge of the reference standard, and whether the timing of the tests was consistent. A previous study developed an evaluation framework based on QUADAS-2 to assess the quality of studies that tested LLM performance on medical questions.^23^ We modified this evaluation framework in our previous work (Supplemental Material 1).^24^

In this evaluation framework, ‘task generation’, ‘conversation structure’, and ‘evaluation’ align with ‘patient selection’, ‘index test’, and ‘reference standard’ in the QUADAS-2,^25^ respectively. Items 2 and 7 align with ‘flow and timing’ in QUADAS-2.

Two authors independently assessed the risk of bias in each study based on the evaluation framework. Any inconsistencies were resolved through discussion.

### Evidence synthesis

The evidence synthesis focuses on GPT-3.5, GPT-4, and Bard.

### Qualitative analyses

We conducted a thorough summary using narrative analysis and descriptive statistics for the studies that were narrative in nature or lacked sufficient data.

### Quantitative analyses

We calculated the accuracy rate using the raw correct and total data from each included study. The calculation rules were as follows: for studies that used a single set of questions for repeated testing, the reported accuracy rate is the average score across all attempts and the total number of questions in that set. For studies that tested with and without optimized prompts, the reported accuracy rate was based on the scores obtained without optimized prompts.

Additionally, we conducted a meta-analysis for LLMs (GPT-3.5, GPT-4, Bard) that were tested in more than two studies.

We employed the *I*² statistic was employed the impact of heterogeneity on the combined results. If significant heterogeneity was detected (*I*² > 50%), a random effects model was utilized; otherwise, a common effects model was utilized. Accuracy was presented with 95% confidence intervals (CIs). The threshold for statistical significance was set at *P* < .05. We conducted meta-regression and subgroup analyses to identify potential sources of heterogeneity and to compare performance across different subgroups. A sensitivity analysis was conducted to evaluate the robustness of the meta-analysis results. We conducted egger test to assess the publication bias. The meta-analysis, sensitivity analyses, and publication bias, were conducted using the ‘metafor’ and ‘meta’ packages in R (version 4.4.0).

## Results

### Literature screening and selection

We collected 3493, 6048, and 6057 articles from WOS, Scopus, and PubMed, respectively. After excluding 4914 duplicate articles and 863 non-English articles, leaving 9821 articles. After excluding 9583 articles completely unrelated to the research topic, 238 remained.

A total of 137 focused on ChatGPT's performance in medical speciality examinations, 46 on medical licensing examinations, 6 on nursing examinations, 6 on pharmacist examinations, and 25 on other medical examinations (eg, university medical entrance examinations and university medical final examinations). Further, 7 articles were dental questions developed by authors themselves. These studies did not meet the inclusion criteria.

**Fig. 1:**
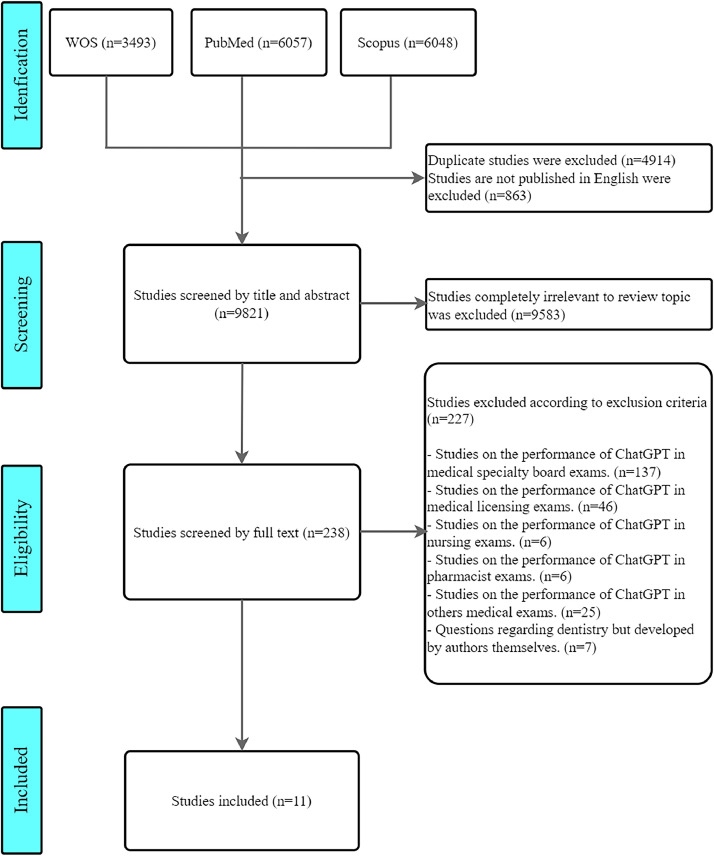
PRISMA (Preferred Reporting Items for Systematic Reviews and Meta-Analyses) flow diagram.

Ultimately, 11 papers were included in this systematic review (Figure 1).^11^, ^12^, ^13^, ^14^, ^15^, ^16^, ^17^, ^18^, ^19^, ^20^, ^21^

### Quality assessment of included studies

In our evaluation framework (Supplemental Material 1), five studies are marked as ‘unclear’, in item 7, indicating that these studies did not report the time when questions were input into the LLMs. Considering that LLMs continuously update their training data,^26^ we believe that if the time span for inputting questions is too long, the LLMs may update their training data during this period, leading to inconsistent responses.

Secondly, in item 10, six studies did not report whether a new chat was used for each question, and one study mentioned grouping 20 questions into a single chat session. We believe that inputting multiple questions into one chat session could potentially cause interference between different questions.

Regarding the items related to evaluators (items 13, 14, 15, and 21), most studies did not provide specific information about the evaluators. However, since the studies we included used multiple-choice questions form dental licensing examinations with standardized answers to test the LLMs, unlike open-ended questions, the absence of multiple evaluators would not impact the quality of the studies.

For the reasons mentioned above, only one study was rated as high risk in both the ‘Index test’ and ‘Flow and Timing’ categories, respectively (Table 3).

**Table 3:** Risk of bias.

Risk of bias				
Study	Patient selection	Index test	Reference standard	Flow and timing
Ohta^11^	Low	Low	Low	Low
Chau^12^	Unclear	Unclear	Low	Unclear
Turunç Oğuzman^13^	Low	Low	Low	Low
Danesh^14^	Unclear	Low	Low	High
Revilla-León^15^	Low	Unclear	Low	Unclear
Yamaguchi^24^	Low	Unclear	Low	Low
Morishita^17^	Low	Unclear	Low	Low
Fuchs^18^	Low	High	Low	Low
Farajollahi^19^	Low	Unclear	Low	Unclear
Danesh^20^	Low	Low	Low	Unclear
Bae^21^	Low	Unclear	Low	Unclear

### General characteristics of included studies

Among the 11 studies, there are 10 articles and 1 correspondence, with the earliest published on 27 June 2023, and the latest on 22 April 2024.

The dental licensing examinations used in these 11 studies include 3 from the United States and 3 from Japan. Additionally, there is one examination each from Turkey, the United Kingdom, the European Union, Switzerland, South Korea, and Iran. Note that one study used dental licensing examinations from both the United States and the United Kingdom.

The studies from Turkey, South Korea, and Japan (3 studies) used examinations in their native languages to test the LLMs, while other non-English-speaking countries translated their examinations into English before testing the LLMs.

Nine studies tested GPT-3.5, 7 studies tested GPT-4, and 3 studies tested Bard. Additionally, one study each tested GPT-3, GPT-4V, and BingChat. The General Characteristics of Included Studies was presented in Supplemental Material 2.

### Qualitative analyses

**Fig. 2:**
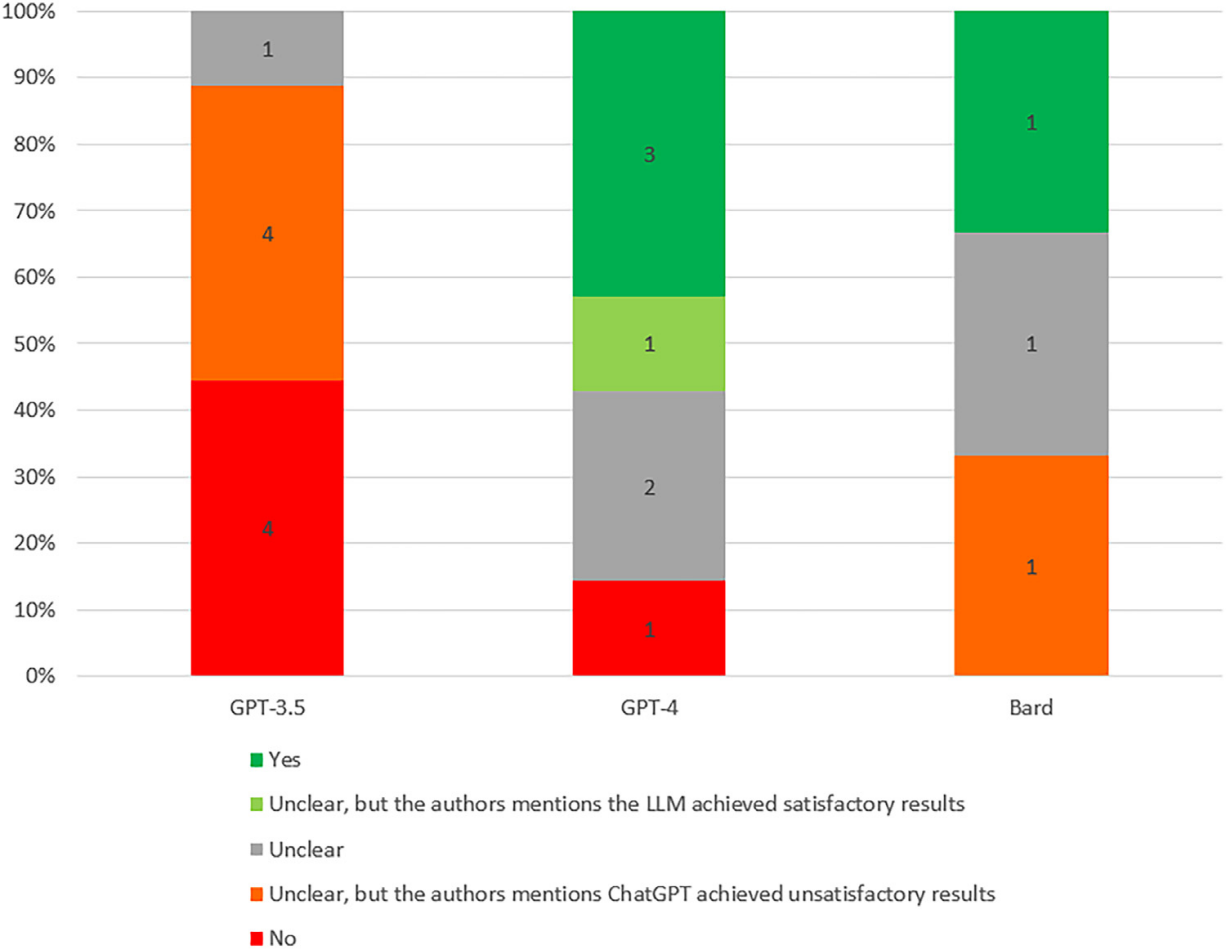
Performance of ChatGPT on passing the dental licensing examination.

Of the 9 studies testing GPT-3.5, 4 indicated that GPT-3.5 failed the examinations, 4 reported that GPT-3.5 achieved unsatisfactory results, and 1 study was unclear. Among the 7 studies testing GPT-4, 3 indicated that GPT-4 passed the examinations, 1 reported that GPT-4 achieved satisfactory results, 1 indicated that GPT-4 failed, and 2 studies were unclear. Regarding the 3 studies on Bard, one reported that Bard passed the examination. Additionally, one study each reported that GPT-4V and GPT-3 failed the examinations (Figure 2).

Six studies used prompts to test LLMs’ performance. However, in 3 Japanese studies and 1 Turkish study, the prompts were only used to help the LLMs understand the task.^11^^,^^13^^,^^16^^,^^17^ In contrast, studies from Swiss and South Korea used prompts to optimize the performance of the LLMs.^18^^,^^21^ The Swiss study compared the performance of LLMs with and without prompts. Before testing, the LLMs were provided with background information about the examination, including its purpose, detailed information about the tests, relevant keywords, and guidelines on the format and answering of questions. With the help of these prompts, the accuracy of GPT-3 and GPT-4 only increased by 3.3% and 2.3%, respectively, compared to without prompts, with significant difference (GPT-3: *P* = .012, GPT-4: *P* = .03).^18^

In the Korean study, the authors modified sentences that could cause ambiguity and added English annotations for Korean and Chinese terms. However, they did not compare the performance of LLMs with and without prompts.^21^

### Meta-analysis

We conducted a meta-analysis on the results of 9 studies testing GPT-3.5, 7 studies testing GPT-4, and 3 studies testing Bard, calculating the integrate accuracy rates for these three LLMs.

**Fig. 3:**
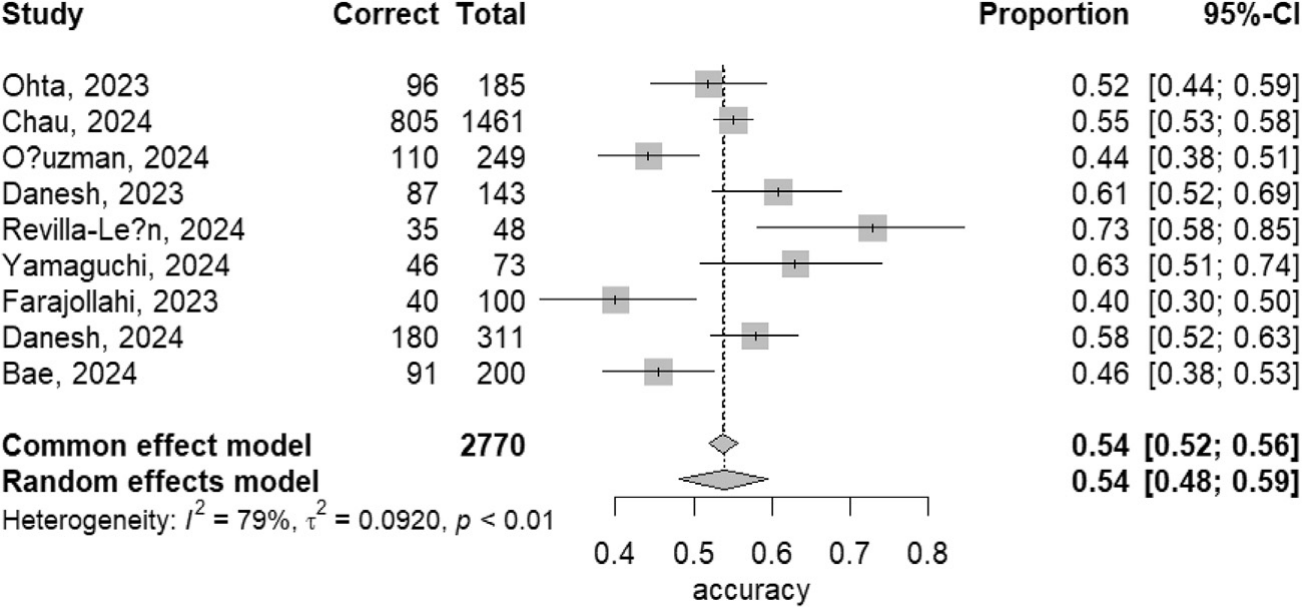
Meta-analysis result of GPT-3.5.

In the results of GPT-3.5, since the *I*² was 79% (*I*² > 50%), a random effects model was used. The integrate accuracy rate for GPT-3.5 was 54% (95% CI: 48%-59%) (Figure 3).

**Fig. 4:**
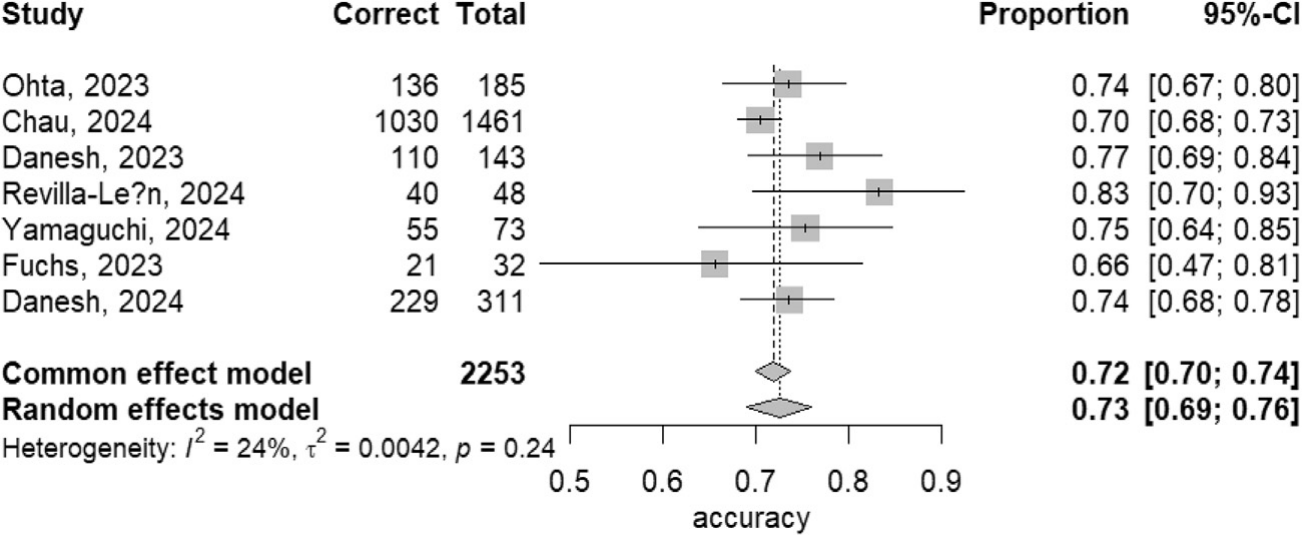
Meta-analysis result of GPT-4.

In the results of GPT-4, since the *I*² was 24% (*I*² < 50%), a common effects model was used. The integrate accuracy rate for GPT-4 was 72% (95% CI: 70%-74%) (Figure 4).

Because only three studies tested Bard, a common effects model was used, despite the *I*² being 91% (*I*² > 50%). The integrated accuracy rate for Bard was 56% (95% CI: 52%-60%) (Supplemental Material 3).

### Subgroup analysis and meta-regression

Since the number of studies for GPT-3.5 and GPT-4 were 9 and 7, respectively, we were able to establish one subgroup with sufficient. The subgroups consisted of studies that used dental licensing examinations from English-speaking countries and those from non-English-speaking countries. As there were only 3 studies on Bard, it could not be included in the subgroup analysis.

In the subgroup analysis for GPT-3.5, a random effects model was used due to the *I*² > 50%. The integrate accuracy rate of GPT-3.5 in non-English-speaking countries’ examinations was 48% (95% CI: 42%-54%), while in English-speaking countries’ examinations, it was 57% (95% CI: 50%-64%). The *P* value indicating significant difference was 0.05, suggesting a borderline level of significance (Supplemental Material 3).

We conducted a meta-regression to further explore the differences across subgroup. In the meta-regression for GPT-3.5, the estimated regression coefficient was 0.46 (*P* = .011). This indicates that whether the examination originated from an English-speaking country was a potential source of heterogeneity and had a significant impact on the accuracy rate of GPT-3.5.

In the subgroup analysis for GPT-4, a random effects model was also used due to the *I*² < 50%. The integrate accuracy rate of GPT-4 in non-English-speaking countries’ dental examinations was 73% (95% CI: 68%-78%), and in English-speaking countries’ dental examinations, it was also 73% (95% CI: 68%-78%). No significant difference was observed (*P* = .97) (Supplemental Material 3).

In the meta-regression for GPT-4, the estimated regression coefficient was 0.02 (*P* = .911). This suggests that whether the examination originated from an English-speaking country was not a potential source of heterogeneity and did not significantly affect the accuracy rate of GPT-4.

### Sensitivity analyses

We conducted a sensitivity analysis on the meta-analysis results of GPT-3.5, GPT-4, and Bard. The results showed that excluding any single study did not affect the integrate accuracy. This confirms that our findings are robust and reliable (Supplemental Material 3).

### Publication bias

We used Egger's test to evaluate publication bias in the studies included in the meta-analyses for GPT-3.5, GPT-4, and Bard. The Egger's test *P* values for the studies included in the meta-analyses were all greater than 0.05 (GPT-3.5: *P* = .094, GPT-4: *P* = .953, Bard: *P* = .530), indicating that no publication bias was observed.

## Discussion

This is the first systematic review and meta-analysis that comprehensively evaluates the performance of LLMs in dental licensing examinations across different countries.

This systematic review included 11 studies, with dental licensing examinations used to test LLMs from 8 countries. Our analysis showed that the integrate accuracy of GPT-3.5 was 54%, and it did not pass any of the dental licensing examinations. GPT-4 performed better than GPT-3.5, achieving an integrate accuracy of 72% and passing more than half of the dental licensing examinations. Bard achieved an integrate accuracy of 56% and passed 1 out of the 3 dental examinations.

We used subgroup analysis and meta-regression to assess whether the dental licensing examinations were from English-speaking countries affect the performance of LLMs. The results indicated that GPT-3.5 performed significantly better on dental licensing examinations from English-speaking countries compared to non-English-speaking countries. GPT-4, however, showed no significant difference. This finding is consistent with previous study on ChatGPT's performance in medical licensing examinations.^24^

We identified several issues regarding the application of LLMs in dental education and clinical diagnostics. Previous study has suggested that for LLMs to be effectively used in medical education and clinical diagnostics, their knowledge accuracy needs to reach 95%.^27^ In this meta-analysis, the integrate accuracy rates for GPT-3.5, GPT-4, and Bard were 54%, 72%, and 56%, respectively – falling significantly short of the 95% threshold. This indicates that the current versions of LLMs are not yet suitable for use in dental education. The attitudes of the authors of the included studies also support our viewpoint. In most of the included studies, even those where LLMs passed the examinations, researchers expressed a pessimistic and cautious attitude towards the accuracy of LLMs’ dental knowledge and the application of LLMs in dental education and clinical decision-making. Four studies indicated that because LLMs generate a significant amount of false, incorrect, or misleading information, they are not currently suitable as educational tools.^13^^,^^16^^,^^18^^,^^20^ Two Studies mentioned that LLMs cannot replace dentists in diagnosis, treatment, or clinical judgment.^11^^,^^17^

Additionally, the performance of LLMs in dental licensing examinations is inferior to their performance in medical licensing examinations, which aligns with findings from a Japanese study.^11^ 54% accuracy rate of GPT-3 and 72% of GPT-4 in dental licensing examinations are both lower than the accuracy rates reported in another meta-analysis on LLMs’ performance in medical licensing examinations (GPT-3.5: 59%, GPT-4: 81%).^24^

We believe the following factors may contribute to the inferior performance of LLMs in dental licensing examinations compared to medical licensing examinations. First, two previous studies analysed the relationship between LLMs’ performance in different medical specialities and the number of publications available in the WOS for each speciality.^28^^,^^29^ The results showed a positive correlation between LLMs’ performance in various medical specialities and the availability of relevant publications online. We found that there are nearly 500,000 publications in the category of Dentistry, Oral Surgery & Medicine in WOS, which is significantly fewer than the 20 million publications in the category of all medical specialities.^30^ Therefore, LLMs may lack sufficient dental-related publications for training, leading to poorer performance.^18^ Second, among the 11 studies included in our research, 8 were from non-English-speaking countries. The study from Iran indicated that the availability of dental publications in non-English languages online is extremely limited.^19^ This poses additional challenges for LLMs when dealing with dental questions from non-English-speaking countries. Our subgroup analysis also showed that LLMs, particularly GPT-3.5, performed significantly worse on examinations from non-English-speaking countries compared to those from English-speaking countries. The inclusion of more non-English-speaking countries in the tests may further decrease LLMs’ performance. Third, the medical field heavily relies on foundational knowledge and has a vast amount of available data. In contrast, dental examinations depend more on visual or practical elements. This presents additional challenges for LLMs.^8^^,^^18^

Previous study has found that due to the nature of LLMs, it often provides detailed and seemingly logical explanations even when they select the wrong answer in medical licensure examinations.^29^^,^^31^^,^^32^ Our study found that this flaw is even more significant in dental licensing examinations. Two included studies mentioned that LLMs provided longer and more detailed explanations for incorrect answers than for correct ones.^14^^,^^20^ Due to the authoritative writing style of LLMs, dental students may find it difficult to recognize errors in the responses, and the more extensive explanations for incorrect answers may reinforce students’ misconceptions. This presents a significant challenge in applying LLMs to dental education.

Compared to medical examinations, dental knowledge, and diagnostics rely more heavily on image interpretation.^8^^,^^18^ However, due to the lack of image recognition capabilities in earlier versions of LLMs, 10 out of the 11 included studies excluded image-based questions. Only one Japanese study reported that GPT-4V achieved an accuracy rate of 35% on 160 image-based questions.^17^ This was the lowest accuracy rate recorded across all studies included in the review. Additionally, a previous study indicated that even the most advanced LLMs, such as GPT-4o, Claude 3 Opus, and Gemini 1.5 Pro, showed significantly lower accuracy on image-based questions compared to nonimage-based ones.^28^ Therefore, we believe that if dental licensing examinations containing image-based questions were used to test LLMs, their accuracy would likely decrease further. We look forward to the development of LLMs with enhanced image recognition capabilities to address this issue.

### Limitation

The publication language was limited in English in this study. As a result, there may be literature published in non-English languages that tested LLMs’ performance in dental licensing examinations from non-English-speaking countries that were not collected.

Additionally, the publication dates for the studies included in this review were restricted to those published before 30 April 2024. Therefore, the performance of the latest LLMs (such as GPT-4o, Claude 3 Sonnet) in dental licensing examinations remains unknown. Previous study reported that GPT-4o performed significantly better than GPT-4 in medical licensing examinations.^28^ This indicates that more advanced LLMs may show even greater potential for use in dental education and clinical diagnostics.

## Conclusions

This systematic review and meta-analysis included 11 studies. GPT-4 achieved an integrate accuracy of 73%, outperforming 54% of GPT-3.5 and 56% of Bard. However, compared to medical licensing examinations, LLMs performed worse and faced greater challenges in dental licensing examinations. The limited number of dental-related publications means that LLMs do not have access to sufficient training data, resulting in worse accuracy rate. Unlike medical fields, dentistry relies more heavily on practical skills and visual images, and LLMs’ poor image recognition capabilities hinder their performance in dental diagnostics. Additionally, LLMs often provide more detailed explanations for incorrect knowledge than for correct information in dental licensing examinations. Given these reasons, we believe that applying LLMs to dental education and clinical diagnosis at this stage is high-risk and inappropriate, presenting more significant challenges than their application in the medical field.

## CRediT authorship contribution statement

**Mingxin Liu:** Conceptualization, Methodology, Software, Validation, Investigation, Data curation, Writing – original draft, Writing – review & editing, Funding acquisition. **Tsuyoshi Okuhara:** Conceptualization, Methodology, Investigation, Writing – original draft, Writing – review & editing, Supervision. **Wenbo Huang:** Methodology, Software, Validation, Data curation, Writing – review & editing. **Atsushi Ogihara:** Methodology, Investigation, Writing – review & editing. **Hikari Sophia Nagao:** Methodology, Data curation, Writing – review & editing. **Hiroko Okada:** Software, Investigation, Writing – review & editing. **Takahiro Kiuchi:** Data curation, Resources, Writing – review & editing.

## Conflict of interest

The authors declare the following financial interests/personal relationships which may be considered as potential competing interests: MINGXIN LIU reports financial support was provided by Japan Society for the Promotion of Science. If there are other authors, they declare that they have no known competing financial interests or personal relationships that could have appeared to influence the work reported in this article.

## Appendix Supplementary materials

Image, application 1

Image, application 2

Image, application 3

Image, application 4

## Footnotes

Supplementary material associated with this article can be found in the online version at doi:10.1016/j.identj.2024.10.014.

## References

[1] OpenAI. ChatGPT. https://chat.openai.com/chat. [Accessed 12 July 2024]

[2] Kung TH, Cheatham M, Medenilla A (2023). Performance of ChatGPT on USMLE: potential for AI-assisted medical education using large language models. PLOS Digit Health.

[3] Yilmaz R., Yilmaz F.G.K. (2023). Augmented intelligence in programming learning: examining student views on the use of ChatGPT for programming learning. Comput Human Behav: Artif Hum.

[4] Biswas S. Role of chatGPT in Law: According to chatGPT. Available at SSRN 4405398. 2023 Mar 30. 10.2139/ssrn.4405398.

[5] Wenzlaff K, Spaeth S. Smarter than humans? Validating how OpenAI’s ChatGPT model explains crowdfunding, alternative finance and community finance. Validating how OpenAI’s ChatGPT Model Explains Crowdfunding, Alternative Finance and Community Finance. Available at SSRN 4302443. 2022 Dec 22. 10.2139/ssrn.4302443.

[6] Alhaidry HM, Fatani B, Alrayes JO, Almana AM, Alfhaed NK. (2023). ChatGPT in dentistry: a comprehensive review. Cureus.

[7] Eggmann F, Weiger R, Zitzmann NU, Blatz MB. (2023). Implications of large language models such as ChatGPT for dental medicine. J Esthet Restor Dent.

[8] Schwendicke F, Samek W, Krois J. (2020). Artificial intelligence in dentistry: chances and challenges. J Dent Res.

[9] Pahadia M. (2023). ChatGPT in dentistry: is it worth the hype?. BDJ In Practice.

[10] Huang H, Zheng O, Wang D (2023). ChatGPT for shaping the future of dentistry: the potential of multi-modal large language model. Int J Oral Sci.

[11] Ohta K, Ohta S. (2023). The performance of GPT-3.5, GPT-4, and bard on the Japanese national dentist examination: a comparison study. Cureus.

[12] Chau RCW, Thu KM, Yu OY, Hsung RT, Lo ECM, Lam WYH. (2024). Performance of generative artificial intelligence in dental licensing examinations. Int Dent J.

[13] Turunç Oğuzman R, Yurdabakan ZZ. (2024). Performance of chat generative pretrained transformer and bard on the questions asked in the dental specialty entrance examination in Turkey regarding bloom's revised taxonomy. Curr Res Dent Sci.

[14] Danesh A, Pazouki H, Danesh K, Danesh F, Danesh A. (2023). The performance of artificial intelligence language models in board-style dental knowledge assessment: a preliminary study on ChatGPT. J Am Dent Assoc.

[15] Revilla-León M, Barmak BA, Sailer I, Kois JC, Att W. (2024). Performance of an artificial intelligence-based chatbot (ChatGPT) answering the European certification in implant dentistry exam. Int J Prosthodont.

[16] Yamaguchi S, Morishita M, Fukuda H (2024). Evaluating the efficacy of leading large language models in the Japanese national dental hygienist examination: A comparative analysis of ChatGPT, Bard, and Bing Chat. J Dent Sci.

[17] Morishita M, Fukuda H, Muraoka K (2024). Evaluating GPT-4V's performance in the Japanese national dental examination: a challenge explored. J Dent Sci.

[18] Fuchs A, Trachsel T, Weiger R, Eggmann F. (2023). ChatGPT's performance in dentistry and allergyimmunology assessments: a comparative study. Swiss Dent J.

[19] Farajollahi M., Modaberi A. (2023). Can ChatGPT pass the “Iranian Endodontics Specialist Board” exam?. Iranian Endod J.

[20] Danesh A, Pazouki H, Danesh F, Danesh A, Vardar-Sengul S. (2024). Artificial intelligence in dental education: ChatGPT's performance on the periodontic in-service examination. J Periodontol.

[21] Bae S-M, Jeon H-R, Kim G-N, Kwak S-H, Lee H-J. (2024). Performance of ChatGPT on the Korean National Examination for Dental Hygienists. J Dent Hyg Sci.

[22] McInnes MDF, Moher D, Thombs BD (2018). Preferred reporting items for a systematic review and meta-analysis of diagnostic test accuracy studies: the PRISMA-DTA Statement [published correction appears in JAMA. 2019;322(20):2026. doi: 10.1001/jama.2019.18307]. JAMA.

[23] Wei Q, Yao Z, Cui Y, Wei B, Jin Z, Xu X (2024). Evaluation of ChatGPT-generated medical responses: a systematic review and meta-analysis. J Biomed Inform.

[24] Liu M, Okuhara T, Chang X (2024). Performance of ChatGPT across different versions in medical licensing examinations worldwide: systematic review and meta-analysis. J Med Internet Res.

[25] Whiting PF, Rutjes AW, Westwood ME (2011). QUADAS-2: a revised tool for the quality assessment of diagnostic accuracy studies. Ann Intern Med.

[26] Heath A. All the news from OpenAI's first developer conference. The Verge. 2023. URL: https://www.theverge.com/2023/11/6/23948619/openai-chatgpt-devday-developer-conference-news. [accessed 20 July 2024]

[27] Levin G, Horesh N, Brezinov Y, Meyer R. (2024). Performance of ChatGPT in medical examinations: a systematic review and a meta-analysis. BJOG.

[28] Liu M, Okuhara T, Dai Z, Huang W, Gu L, Okada H, Furukawa E, Kiuchi T (2024). Evaluating the Effectiveness of advanced large language models in medical Knowledge: A Comparative study using Japanese national medical examination. Int J Med Inform.

[29] Haze T, Kawano R, Takase H, Suzuki S, Hirawa N, Tamura K. (2023). Influence on the accuracy in ChatGPT: differences in the amount of information per medical field. Int J Med Inform.

[30] Clarivate. Web of science core collection. https://clarivate.com/products/scientific-and-academic-research/research-discovery-and-workflow-solutions/webofscience-platform/web-of-science-core-collection/. [accessed 18 June 2024]

[31] Roos J, Kasapovic A, Jansen T, Kaczmarczyk R. (2023). Artificial intelligence in medical education: comparative analysis of ChatGPT, Bing, and medical students in Germany. JMIR Med Educ.

[32] Shang L, Xue M, Hou Y, Tang B. (2023). Can ChatGPT pass China's national medical licensing examination?. Asian J Surg.

