# Supplementary material for: Large Language Models in Dental Licensing Examinations: Systematic Review and Meta-Analysis
Source: Int Dent J. 2024 Nov 12;75(1):213–22. doi: 10.1016/j.identj.2024.10.014 (PMC11806300; doi:10.1016/j.identj.2024.10.014)
Supplement: Supplementary file 3 [file mmc3.pptx]

## Slide 1
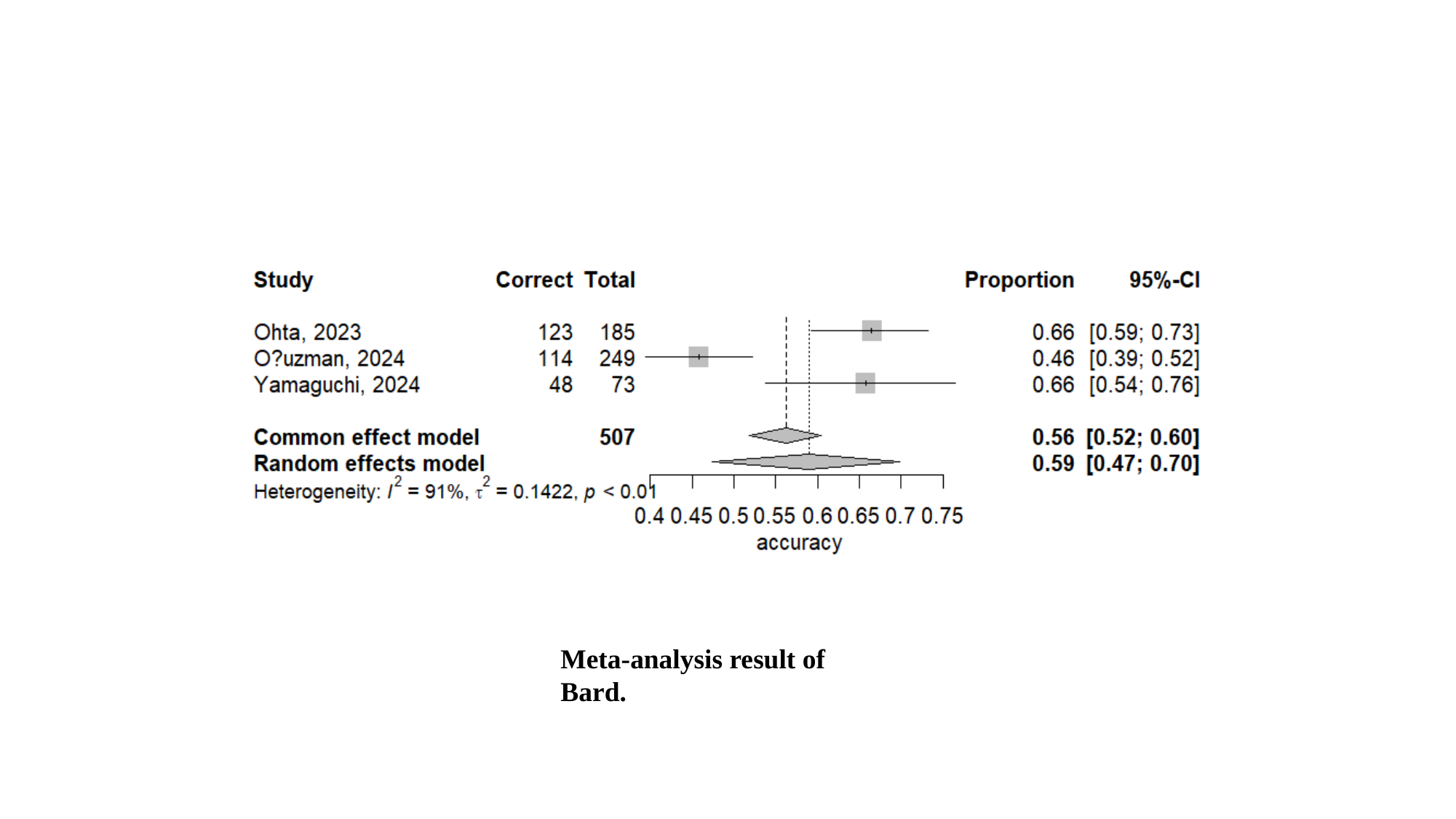

Meta-analysis result of Bard.

## Slide 2
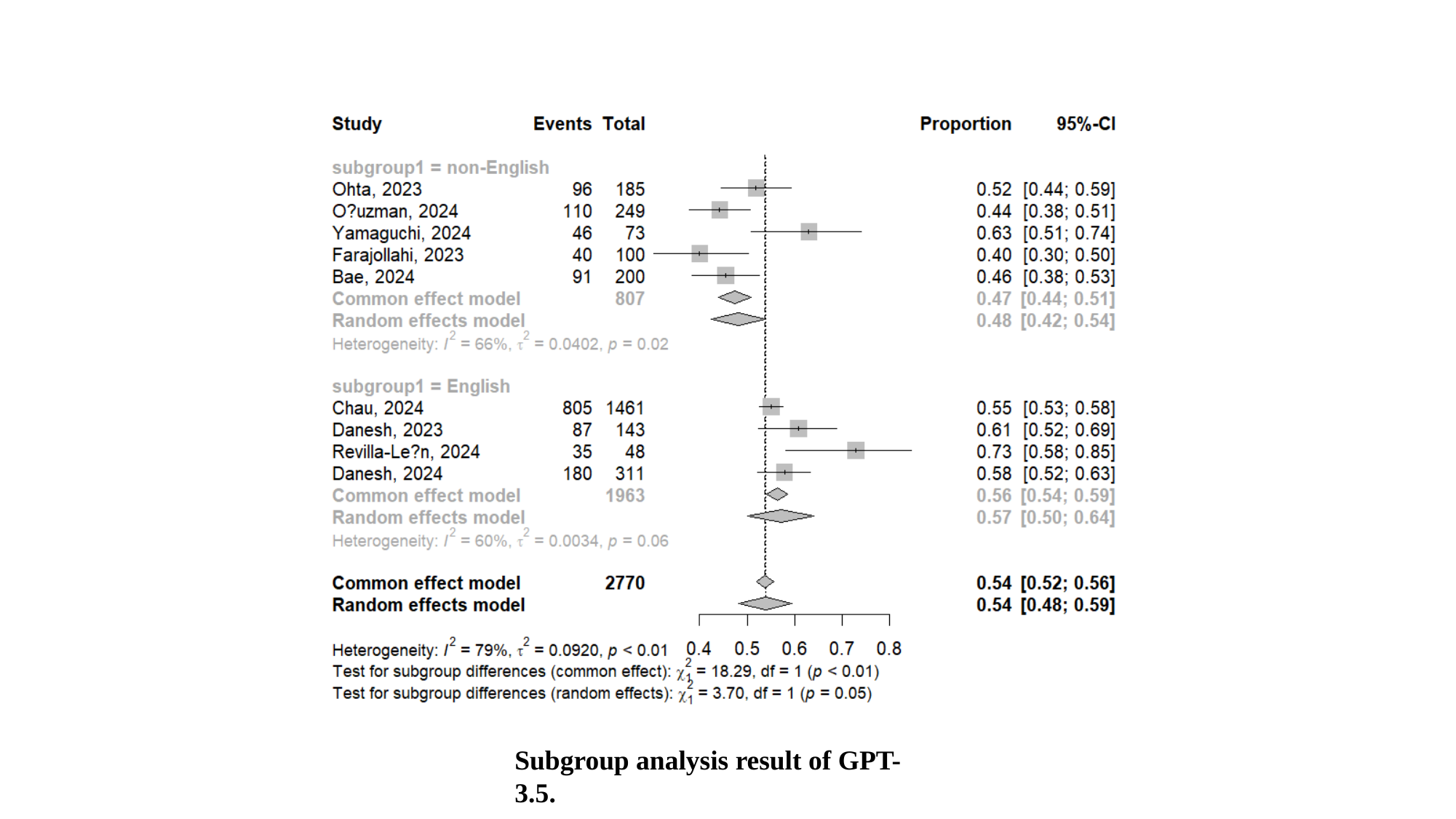

Subgroup analysis result of GPT-3.5.

## Slide 3
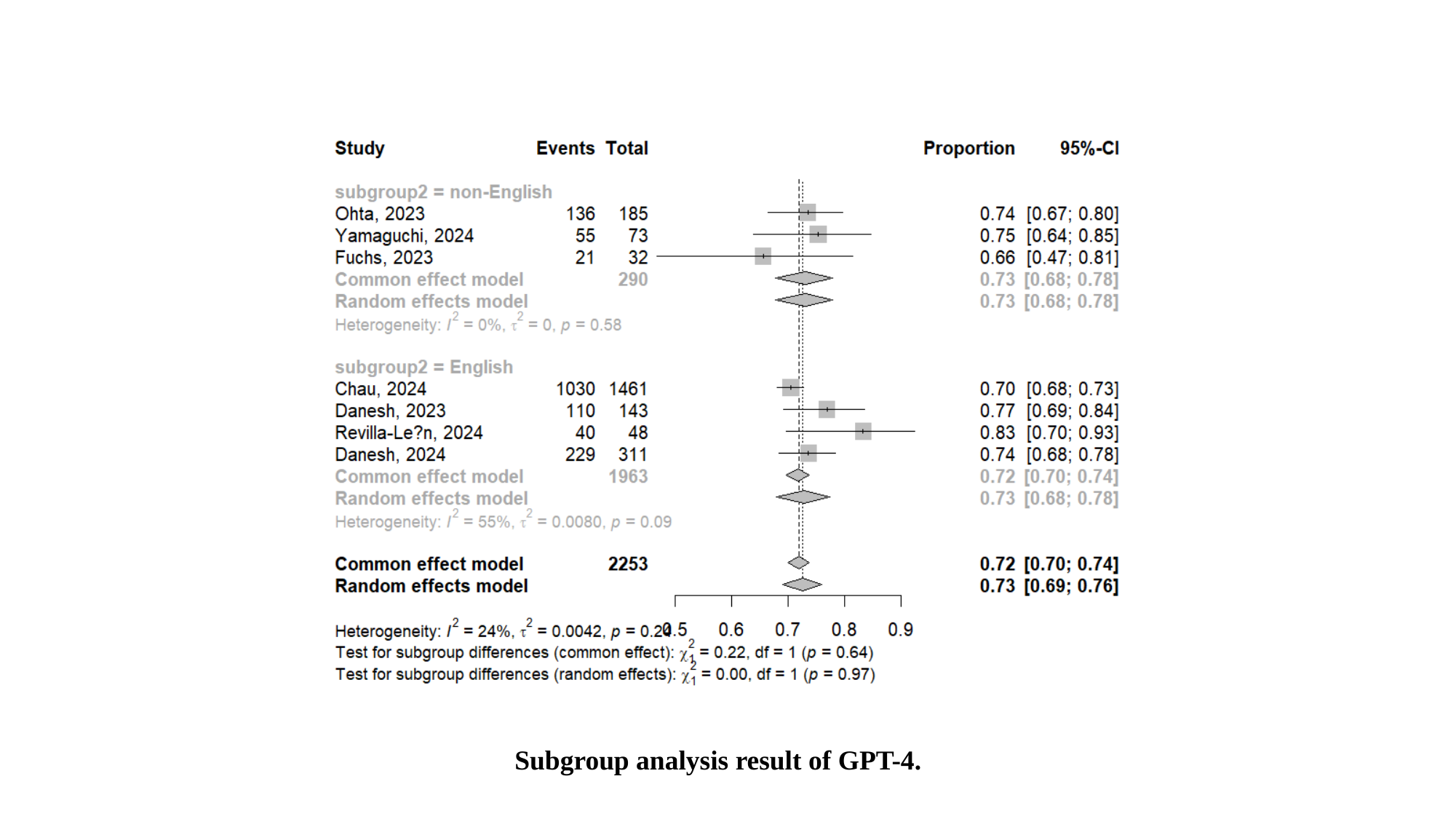

Subgroup analysis result of GPT-4.

## Slide 4
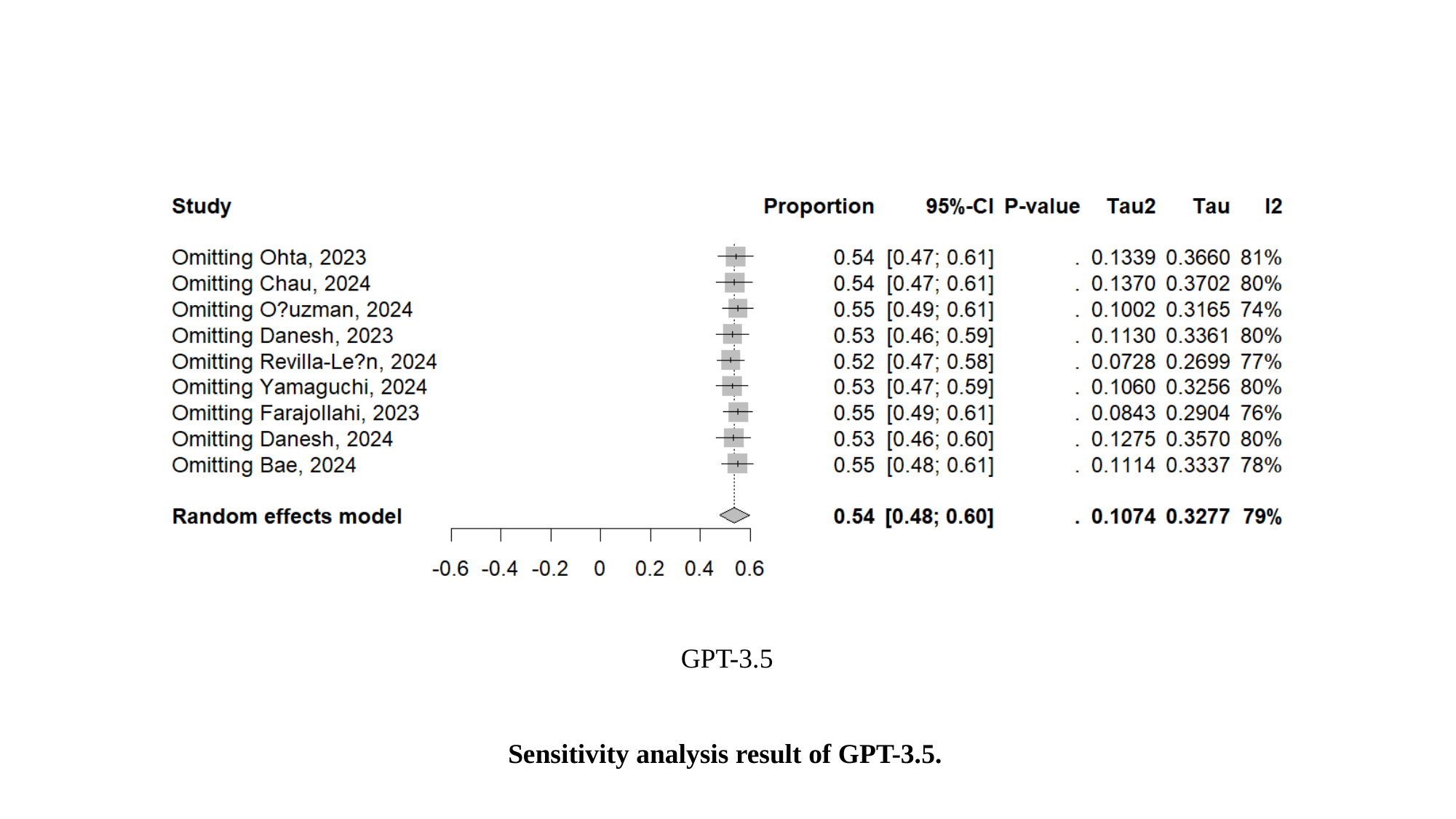

GPT-3.5
Sensitivity analysis result of GPT-3.5.

## Slide 5
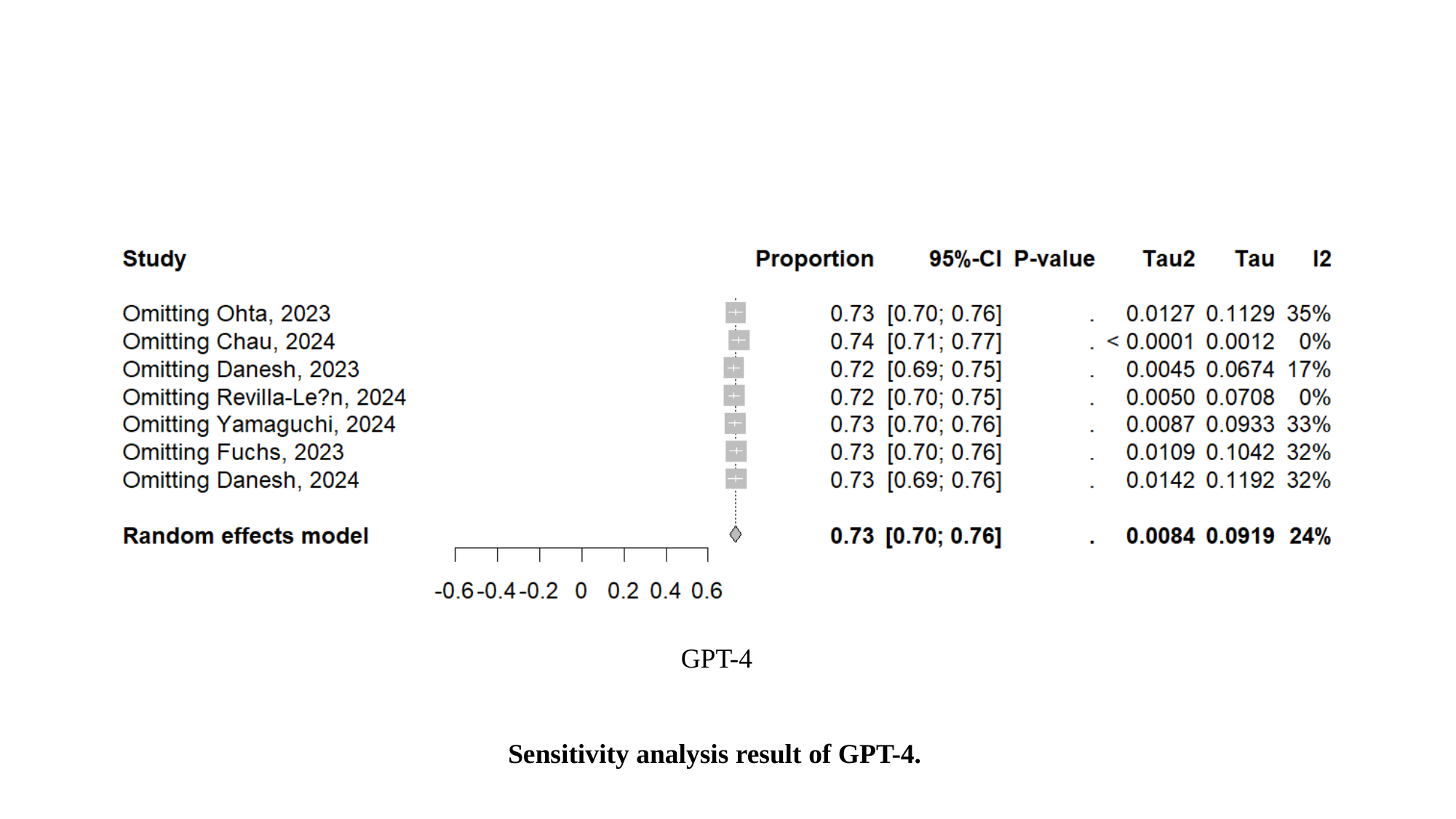

GPT-4
Sensitivity analysis result of GPT-4.

## Slide 6
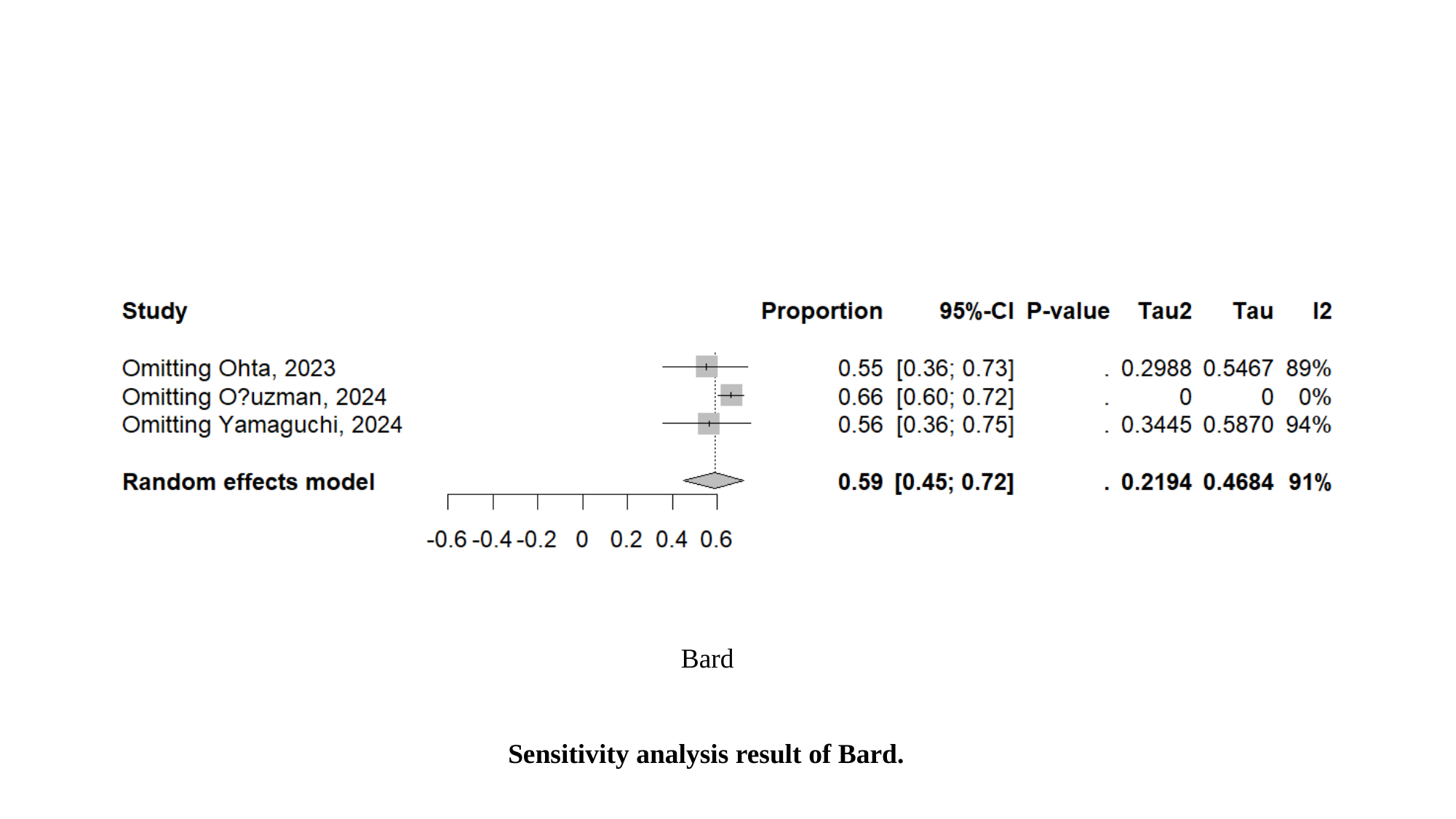

Bard
Sensitivity analysis result of Bard.
